# Supplementary material for: A bacterial toxin-antitoxin system involved in an unusual response to genotoxic stress
Source: EMBO Rep. 2025 Aug 18;26(18):4532–62. doi: 10.1038/s44319-025-00545-y (PMC12457610; doi:10.1038/s44319-025-00545-y)
Supplement: Supplementary file 1 — Appendix [file 44319_2025_545_MOESM1_ESM.pdf]

A bacterial toxin-antitoxin system involved in an unusual response to genotoxic stress

Jordan D. Lin[1†], Beth Nicholson[2], Alexander W. Ensminger[1,2,*]

[1] Department of Molecular Genetics, University of Toronto, Toronto, Ontario, Canada
[2] Department of Biochemistry, University of Toronto, Toronto, Ontario, Canada
*To whom correspondence should be addressed. Email: alex.ensminger@utoronto.ca
[†]Present address: Jordan D. Lin, Department of Medicine, Stanford University, Stanford, CA, USA

TABLE OF CONTENTS

Appendix Table S1……………………………………………………………………………….2

Appendix Figure S1………………………………………………………………………………...3

Appendix Figure S2………………………………………………………………………………...4

Appendix Table S1. SNPs detected across *L. pneumophila* TA deletion strains.

**Strain**	**Deleted genes**	**SNP**	**Outcome**	**Mutated locus**	**Function**	**Upstream gene**	**Downstream gene**
ΔTA1	lpg0488-0489						
ΔTA2	lpg1604-1605	A > G	V > A	tspO	tryptophan rich sensory protein	lpg0210	phrB
ΔTA3	lpg1934-1935						
ΔTA4	lpg2368-2370						
ΔTA5	lpg2377-2380						
ΔTA6	lpg2914-2915	G > A	C > Y	lpg2621	acid phosphatase	lpg2620	lpg2622
ΔTA7	lpg2920-2921						
Δ7TA-1	all 7 systems	C > T		intergenic		lpg2019	oruR
Δ7TA-2	all 7 systems	T > C	silent	lpxB	lipid-A-disaccharide synthase	lpxD	lpg2946

Appendix Figure S1. Enhanced survival is cell extrinsic and conferred through cell-cell contact

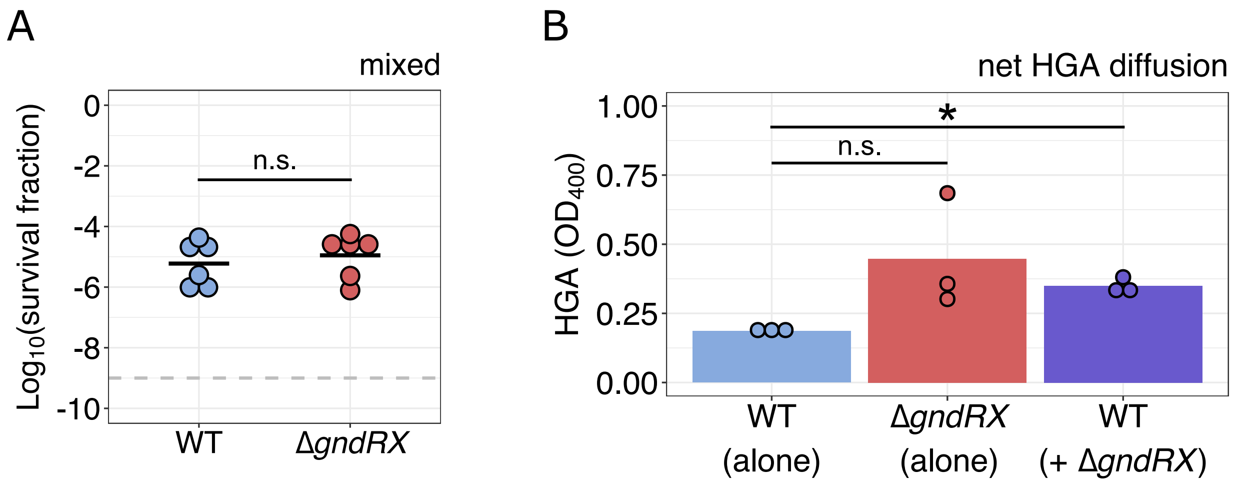

(A) Time-kill assay using permeabilized transwell plate inserts (0.1 μm membrane) that restore cell mixing. Data are from n=3 biological replicates performed in duplicate (each strain with or without the *lux* cassette) and survival after 72 hr treatment with ciprofloxacin is shown. (B) Quantification of HGA diffusion across a 0.1 μm transwell membrane. HGA production was measured when both compartments contained either wild-type or Δ*gndRX* cells, or in a compartment containing wild-type cells when the other compartment contained Δ*gndRX* cells. Data are from n=3 biological replicates after 72 hr treatment with ciprofloxacin. Data information: In (A-B), bar indicates the mean and statistical hypothesis testing was performed with the Welch's t-test (n.s. = not significant; * = $p<0.05$). The limit of detection on all applicable plots is indicated with a dashed grey line.

Appendix Figure S2. GndRX appears to shift the cell from a state of dormancy to death during stress

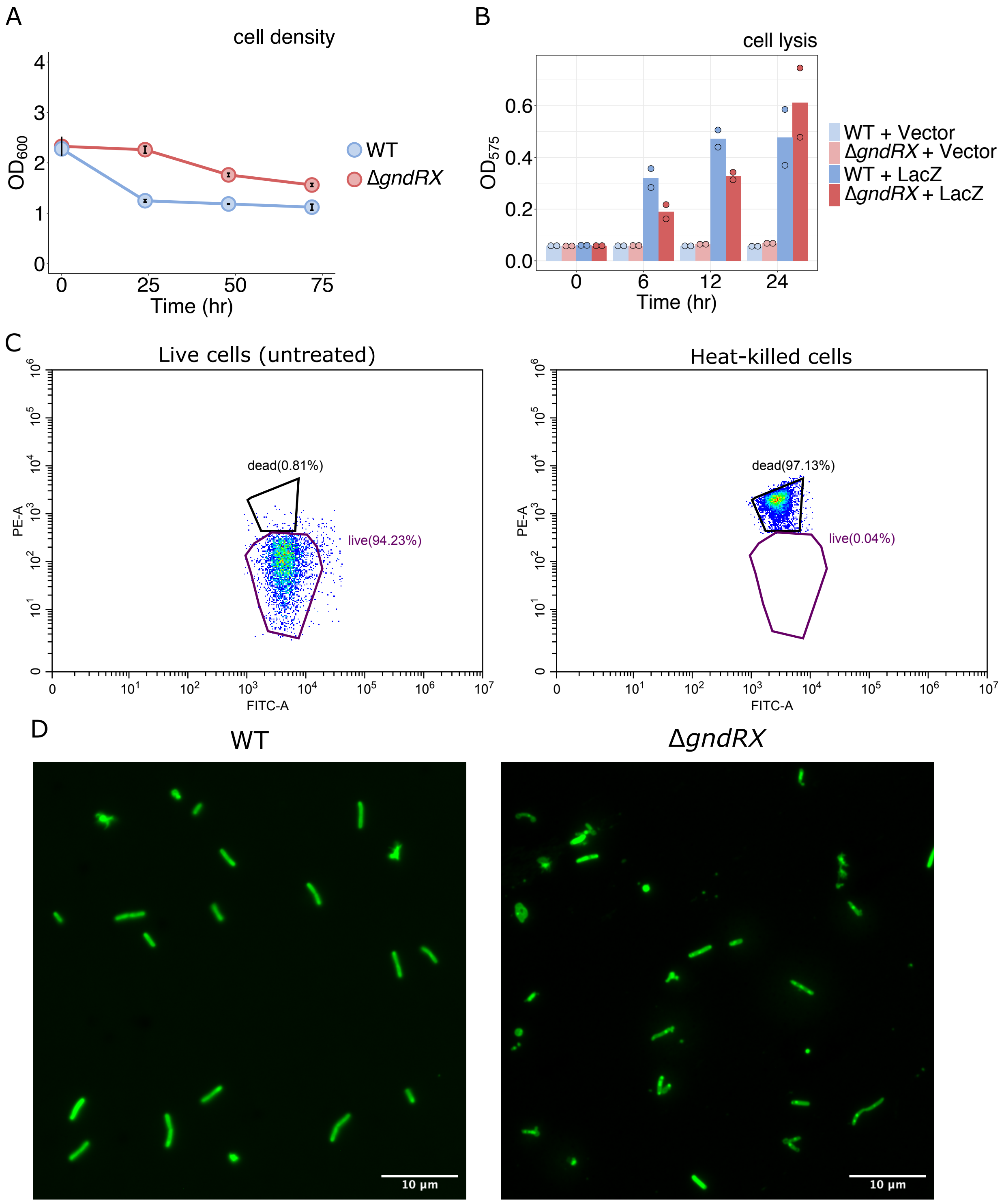

(A) Culture turbidity measurements for the wild-type and Δ*gndRX* strains during treatment with ciprofloxacin (data are mean ± SEM, n=2 biological replicates). At each time point, cells were washed 3x with PBS and absorbance as measured in a cuvette using a spectrophotometer. (B) Cell lysis assay quantifying free LacZ protein in the culture supernatant during treatment with ciprofloxacin for wild-type and Δ*gndRX* strains carrying either an empty vector control (pJB1806) or producing LacZ (pJB1806::*lacZ*). LacZ expression was induced with IPTG (500 µM) for 24 hr, concurrent with genotoxic stress. The culture supernatant was subsequently harvested and supplemented with the LacZ substrate CPRG (20 µg/mL). Colorimetric changes produced by LacZ activity were then measured using a spectrophotometer. Data are from n=2 biological replicates. (C) Flow cytometry quantification using Live/Dead staining for untreated and heat-killed (99°C for 10 min) wild-type populations used as gating controls (10,000 events are displayed; data are representative of n=3 biological replicates) (D) Microscopy images of SYTO9 stained wild-type and Δ*gndRX L. pneumophila* cells after 24 hr ciprofloxacin treatment from a representative flow cytometry experiment. Scale bar is 10 µm. Data information: In A, data are presented as the mean (averaged for clarity) ± SEM. In B, bar indicates the mean.
