# Supplementary material for: A bacterial toxin-antitoxin system involved in an unusual response to genotoxic stress
Source: EMBO Rep. 2025 Aug 18;26(18):4532–62. doi: 10.1038/s44319-025-00545-y (PMC12457610; doi:10.1038/s44319-025-00545-y)
Supplement: Supplementary file 11 — Expanded View Figures [file 44319_2025_545_MOESM11_ESM.pdf]

Expanded View Figures

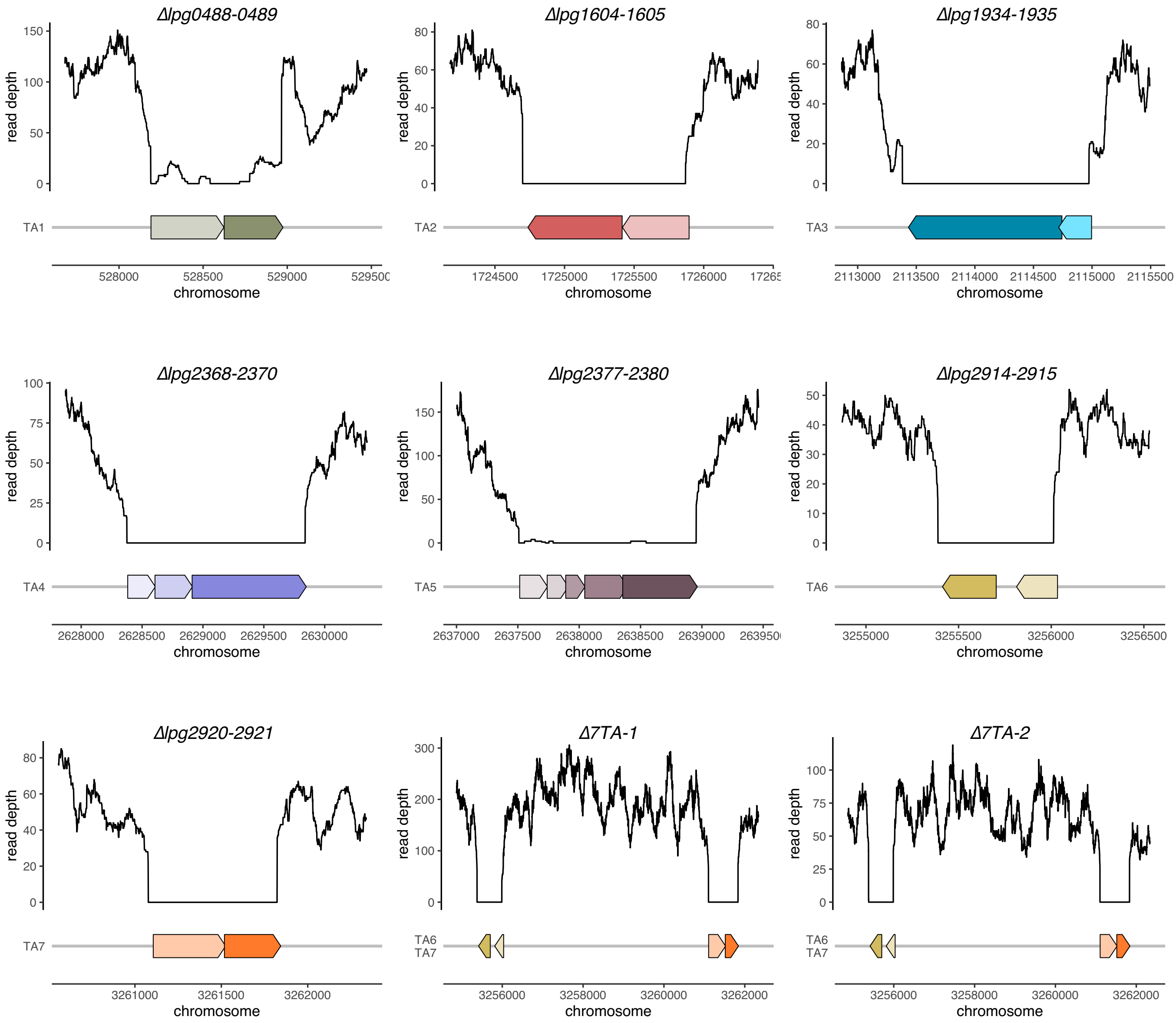

Figure EV1. Genomic sequencing confirms TA system deletions.

Sequencing read coverage of the *L. pneumophila* single and pan-TA deletion strains at the chromosomal locus of each TA system. Reads were aligned to a reference genome (Refseq ID: GCF_001941585.1) to confirm the fidelity of the edits. For the Δ7TA-1 and Δ7TA-2 strains, two TA loci are shown as an example. SNPs detected after sequencing are shown in Appendix Table S1.

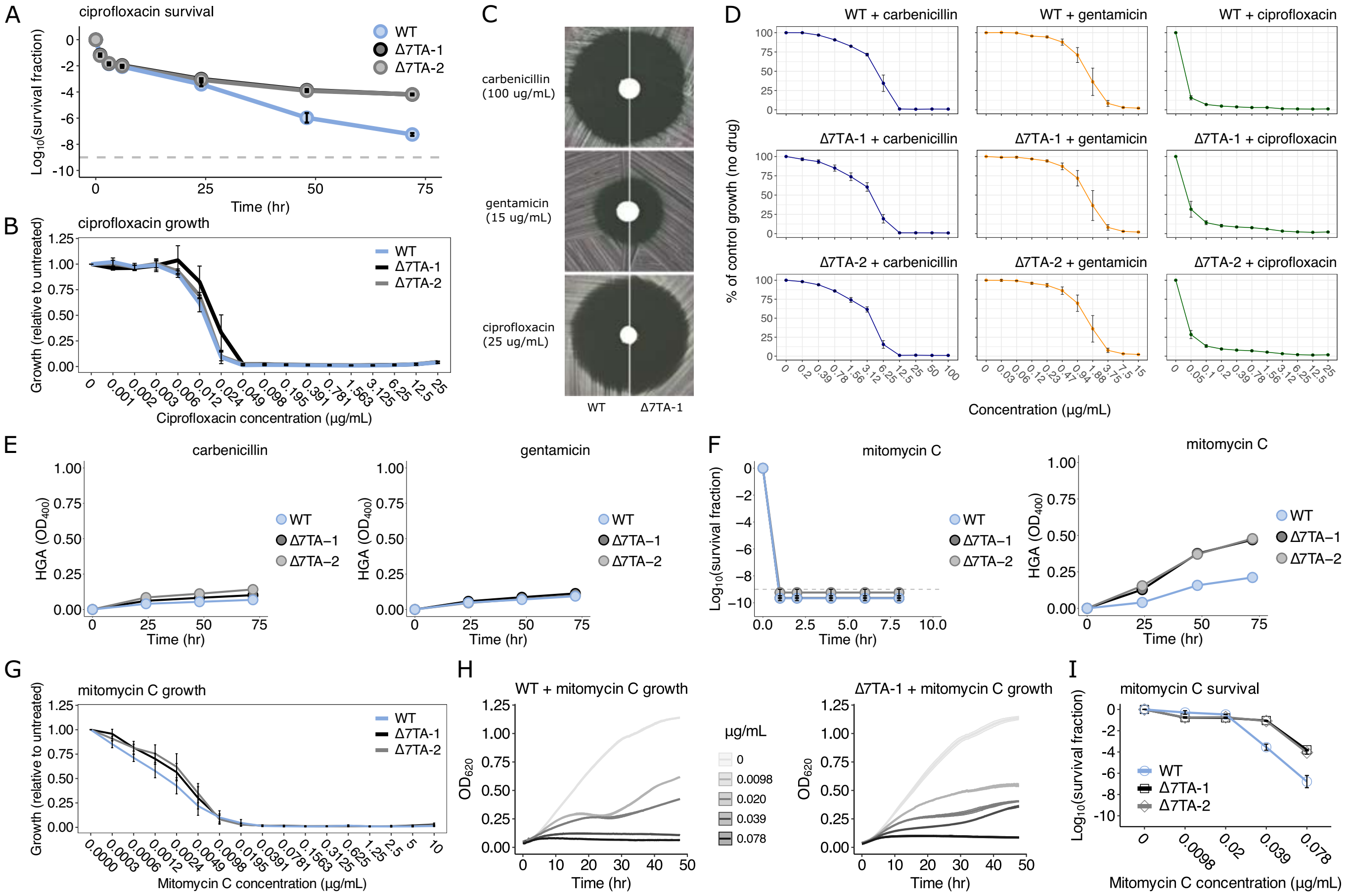

Figure EV2. Antibiotic susceptibility is similar for the *L. pneumophila* wild-type and Δ7TA strains.

(**A**) Time-kill assay measuring survival during treatment with ciprofloxacin ($n = 3$ biological replicates). (**B**) Ciprofloxacin MIC measurements for the wild-type, Δ7TA-1 and Δ7TA-2 strains ($n = 4$ biological replicates). (**C**) Disk diffusion assays for wild-type *L. pneumophila* and the Δ7TA-1 strain treated with carbenicillin, gentamicin, and ciprofloxacin (data are representative of $n = 2$ biological replicates). (**D**) Dose–response curves for wild-type (WT) *L. pneumophila* and two lineages of the Δ7TA strain treated with the antibiotics carbenicillin, gentamicin, and ciprofloxacin ($n = 2$ biological replicates). (**E**) HGA production by the wild-type and Δ7TA strains during treatment with carbenicillin and gentamicin (representative experiments are shown; $n = 2$ biological replicates). (**F**) Time-kill assay (left) and HGA quantification (right) of the wild-type and Δ7TA strains during treatment with mitomycin C. Survival data are from $n = 2$ biological replicates and HGA production from a representative experiment is shown ($n = 2$ biological replicates). (**G**) Mitomycin C MIC measurements for the wild-type, Δ7TA-1 and Δ7TA-2 strains ($n = 4$ biological replicates). (**H**) Dose–response curves for wild-type *L. pneumophila* and the Δ7TA-1 strain treated with mitomycin C at the indicated concentrations ($n = 2$ biological replicates). (**I**) Survival is shown after 48 h treatment with mitomycin C at the indicated concentrations for the wild-type, Δ7TA-1 and Δ7TA-2 strains ($n = 2$ biological replicates). Data information: In (**A**, **B**, **D**, **F–I**), data are presented as the mean (averaged for clarity) ± SEM. The limit of detection on all applicable plots is indicated with a dashed gray line.

 © The Author(s)

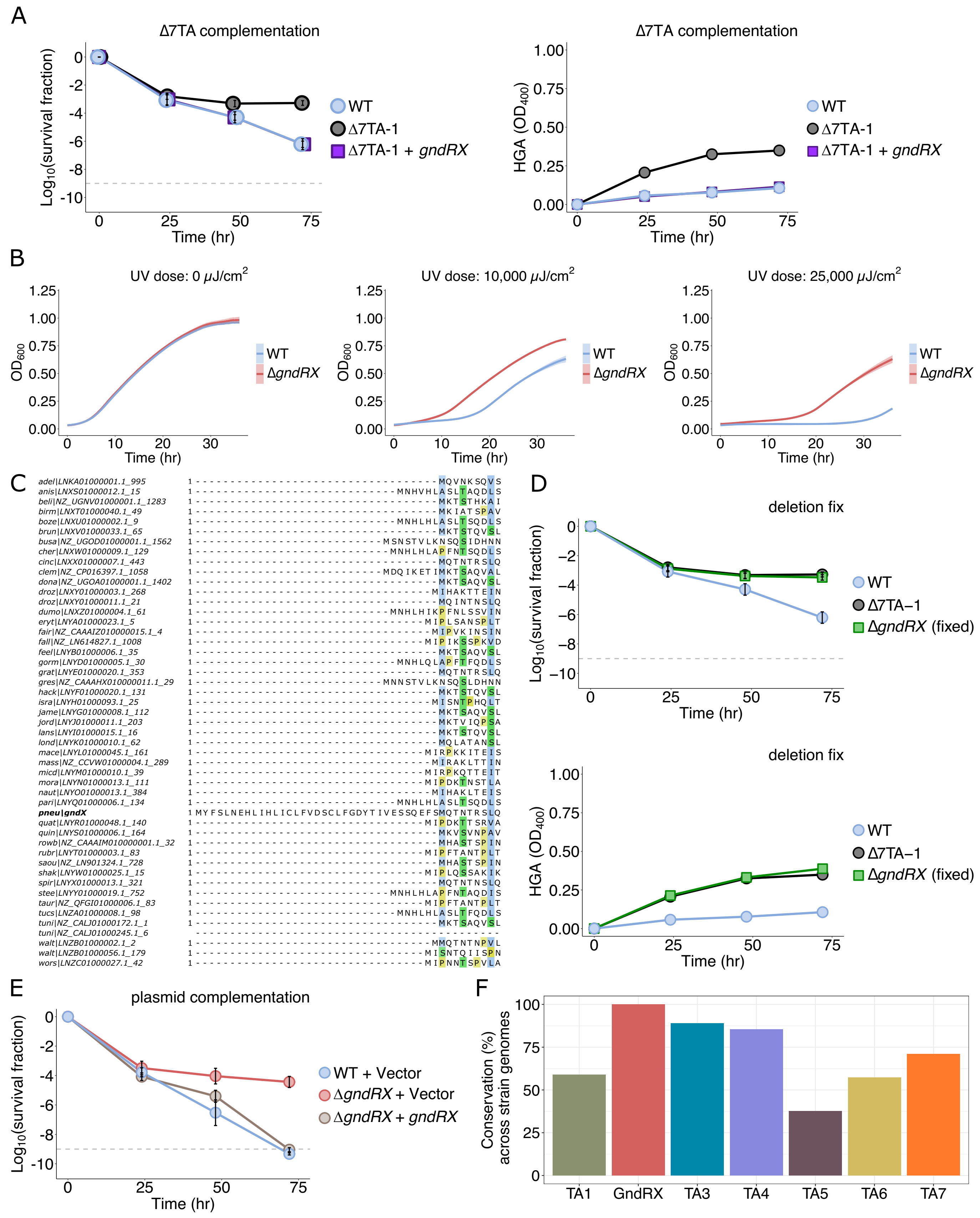
A
Δ7TA complementation
Log10(survival fraction)
Time (hr)
WT
Δ7TA-1
Δ7TA-1 + gndRX
Δ7TA complementation
HGA (OD400)
Time (hr)
WT
Δ7TA-1
Δ7TA-1 + gndRX
B
UV dose: 0 μJ/cm2
UV dose: 10,000 μJ/cm2
UV dose: 25,000 μJ/cm2
OD600
Time (hr)
WT
ΔgndRX
C
D
deletion fix
Log10(survival fraction)
Time (hr)
WT
Δ7TA–1
ΔgndRX (fixed)
deletion fix
HGA (OD400)
Time (hr)
WT
Δ7TA–1
ΔgndRX (fixed)
E
plasmid complementation
Log10(survival fraction)
Time (hr)
WT + Vector
ΔgndRX + Vector
ΔgndRX + gndRX
F
Conservation (%) across strain genomes
TA1
GndRX
TA3
TA4
TA5
TA6
TA7

Figure EV3. The *gndRX* locus is responsible for the genotoxic stress response phenotype.

(**A**) Ciprofloxacin time-kill assay (left) and HGA quantification (right) to test the chromosomal complementation of the *gndRX* locus in the Δ7TA background. The Δ*gndRX* chromosomal lesion was repaired with a linear PCR product to restore the wild-type *gndRX* sequence and confirmed with Sanger sequencing. Survival data are from $n = 2$ biological replicates and HGA production from a representative experiment is shown ($n = 2$ biological replicates). (**B**) Growth curves of wild-type and Δ*gndRX* strains following UV irradiation at the indicated doses (data are representative of $n = 3$ biological replicates). (**C**) Multiple sequence alignment (MUSCLE) of GndX homologs found in *Legionella* species. *L. pneumophila* GndX is shown in bold. Residues conserved in at least 10% of proteins are colored with the Clustal X color scheme as implemented in Jalview. (**D**) Ciprofloxacin time-kill assay (top) and HGA quantification (bottom) for the wild-type, Δ7TA-1, and Δ7TA-1 (fixed Δ*gndRX* deletion) strains. The Δ*gndRX* chromosomal lesion in the Δ7TA-1 strain was repaired with a linear PCR product to restore the upstream 105 bp sequence that was misannotated as part of the *gndX* gene. The edit was confirmed with Sanger sequencing. Survival data are from $n = 2$ biological replicates and HGA production from a representative experiment is shown ($n = 2$ biological replicates). (**E**) Time-kill assay for plasmid complementation of *gndRX* in the Δ*gndRX* background. *L. pneumophila* strains carrying a plasmid (pNT562) with the *gndRX* sequence (without the misannotated 105 bp upstream region) or an empty vector control were treated with ciprofloxacin in the presence of kanamycin for plasmid maintenance. Survival data are from $n = 2$ biological replicates. (**F**) Bar chart of TA system conservation across 117 *L. pneumophila* strains with complete genomes in the NCBI Refseq database (Dataset EV1). Data information: In (**A**, **B**, **D**, **E**), data are presented as the mean (averaged for clarity) ± SEM. The limit of detection on all applicable plots is indicated with a dashed gray line.

© The Author(s)

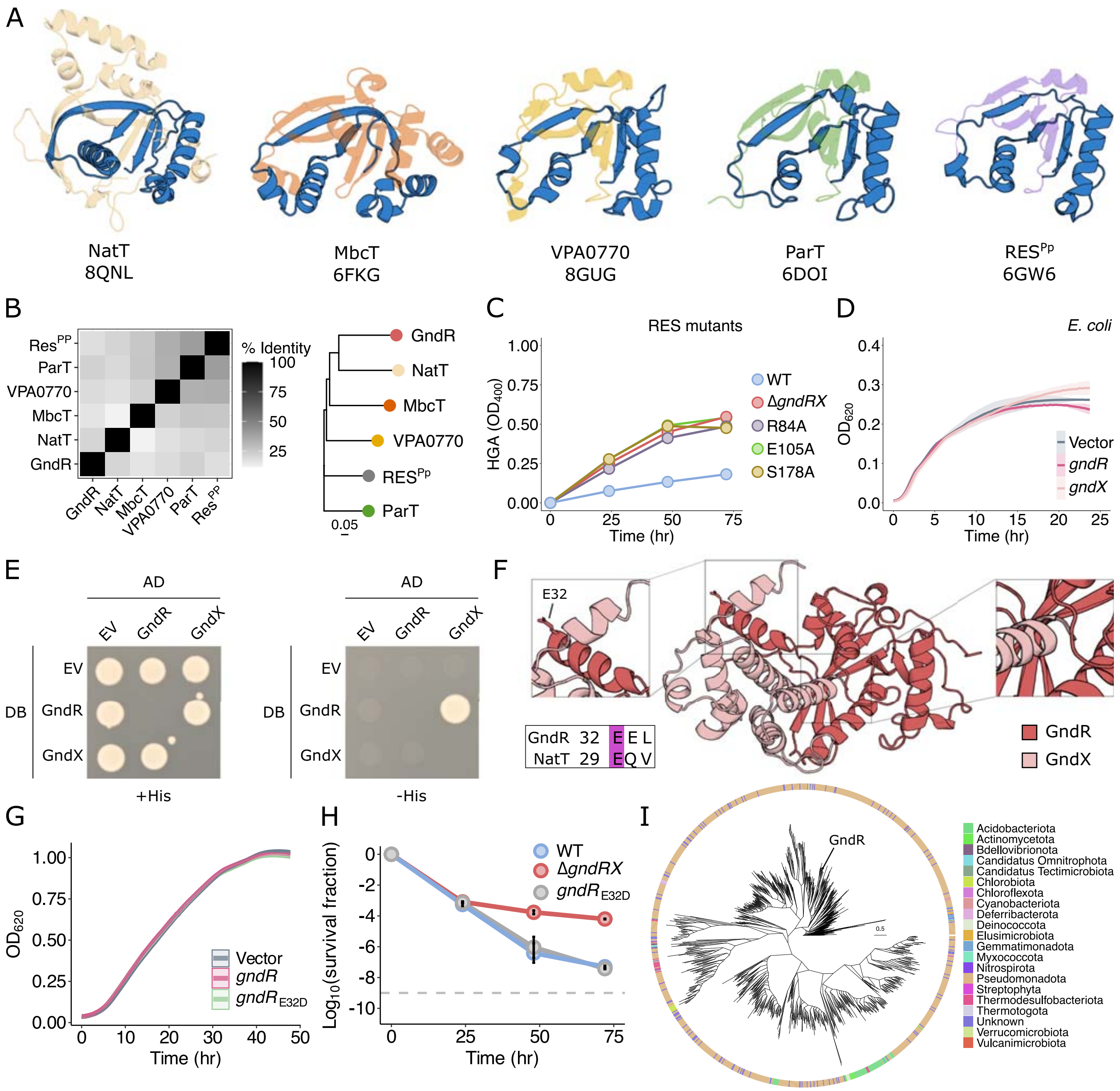
A
NatT
8QNL
MbcT
6FKG
VPA0770
8GUG
ParT
6DOI
RESPp
6GW6
B
% Identity
GndR
NatT
MbcT
VPA0770
RESPp
ParT
0.05
C
RES mutants
HGA (OD400)
Time (hr)
WT
ΔgndRX
R84A
E105A
S178A
D
E. coli
OD620
Time (hr)
Vector
gndR
gndX
E
AD
EV GndR GndX
DB
EV
GndR
GndX
+His
-His
F
E32
GndR 32 E E L
NatT 29 E Q V
GndR
GndX
G
OD620
Time (hr)
Vector
gndR
gndRE32D
H
Log10(survival fraction)
Time (hr)
WT
ΔgndRX
gndRE32D
I
GndR
Acidobacteriota
Actinomycetota
Bdellovibrionota
Candidatus Omnitrophota
Candidatus Tectimicrobiota
Chlorobiota
Chloroflexota
Cyanobacteriota
Deferribacterota
Deinococcota
Elusimicrobiota
Gemmatimonadota
Myxococcota
Nitrospirota
Pseudomonadota
Streptophyta
Thermodesulfobacteriota
Thermotogota
Unknown
Verrucomicrobiota
Vulcanimicrobiota

Figure EV4. GndRX has remote homology to RES-Xre TA systems but non-canonical activity.

(**A**) Crystal structures of NatT, MbcT, VPA0770, ParT, and RES^{Pp}. The predicted RES domains in each protein are colored in blue. (**B**) Multiple sequence alignment (MUSCLE) similarity matrix and gene tree of GndR with the NatT, MbcT, VPA0770, ParT, and RES^{Pp} toxins. (**C**) HGA production from *L. pneumophila* strains containing substitutions for individual R-E-S residues and treated with ciprofloxacin (a representative experiment is shown; $n = 3$ biological replicates). (**D**) Growth curves of *E. coli* expressing *gndR* or *gndX* compared with an empty vector (EV; pBAD33) control ($n = 2$ biological replicates). Expression was induced with 0.2% arabinose. (**E**) Yeast two-hybrid assay of GndRX protein-protein interactions (data are representative of $n = 2$ biological replicates). Constructs cloned with fusions to the activating domain (AD) or DNA-binding domain (DB) of the GAL4 transcription factor are indicated. Control experiments were performed in the presence of histidine (+His) and interaction experiments were performed in the absence of histidine (-His). (**F**) AlphaFold2 predicted interaction complex of GndR-GndX. Insets show the protein regions involved in the interaction (left) and putative regulatory site (right). A multiple sequence alignment between GndR and NatT is shown to highlight the conserved E32 residue in GndR (also shown in left inset). (**G**) Growth curves of *L. pneumophila* expressing *gndR*, $gndR_{E32D}$, or an empty vector control (pNT562). Expression was induced with IPTG (100 μM). Data are from $n = 3$ biological replicates. (**H**) Ciprofloxacin time-kill assay comparing wild-type, Δ*gndRX*, or a strain with the chromosomal mutation $gndR_{E32D}$. Survival data are from $n = 3$ biological replicates. (**I**) Phylogeny of GndR protein homologs detected in the UniProtKB database (Dataset EV2). Sequences were clustered at 80% similarity using MMseqs2 and a representative sequence was used for each cluster. Taxonomic assignment was performed with MMseqs2 using the lowest common ancestor (LCA) protocol. For each representative sequence, the LCA phylum is displayed as the color of the surrounding ring. The scale bars in both trees denotes substitutions per site. Data information: In (**D**, **G**, **H**), data are presented as the mean (averaged for clarity) ± SEM. The limit of detection on all applicable plots is indicated with a dashed gray line.

 © The Author(s)

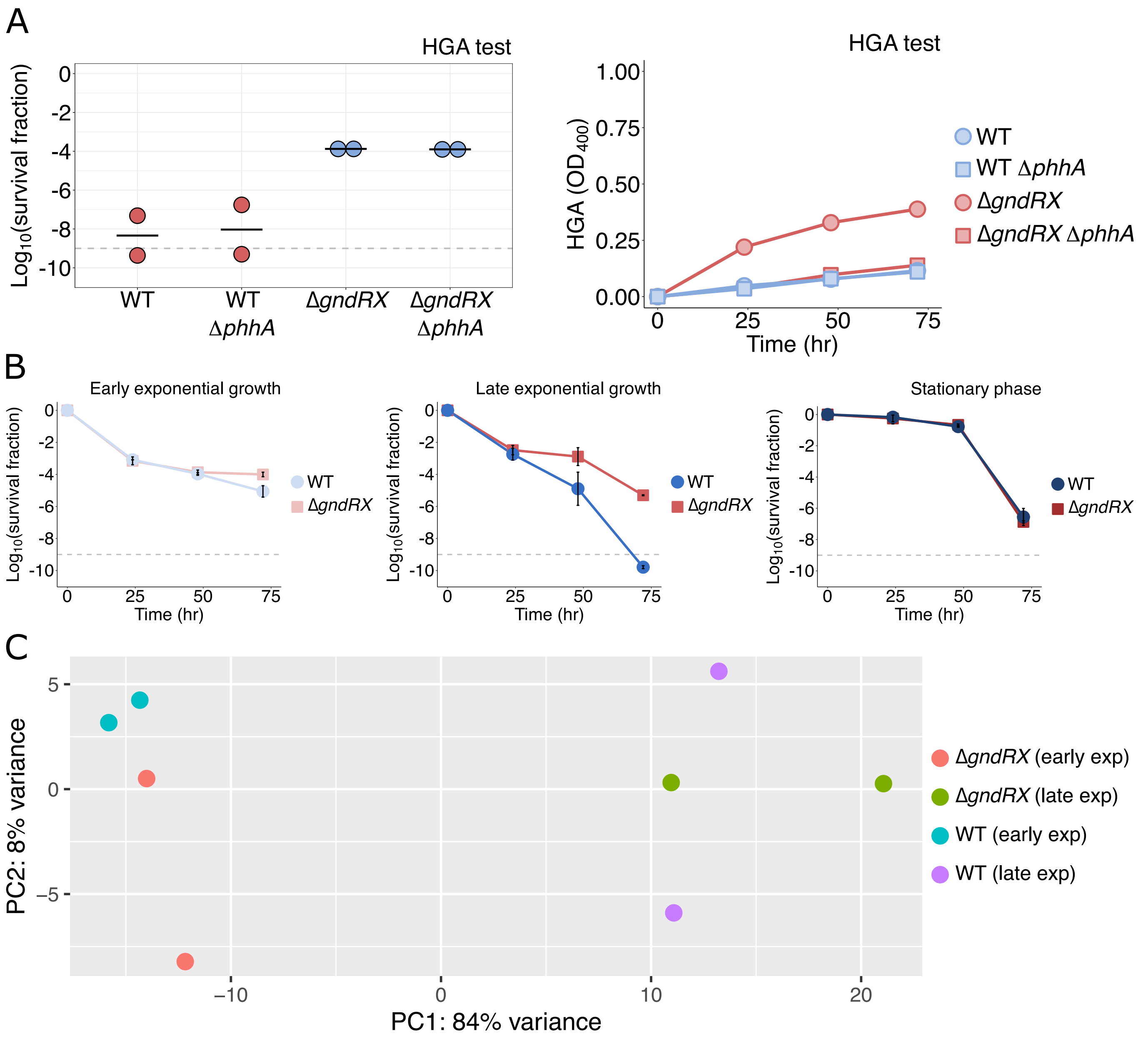

Figure EV5. The stress survival phenotype is HGA-independent but growth phase-dependent.

(**A**) Ciprofloxacin time-kill assay (left) and HGA quantification (right) of wild-type and Δ*gndRX* strains comparing the effect of deleting *phhA* and thereby abrogating HGA biosynthesis. The chromosomal *phhA* locus was deleted using the MazF recombineering technique and confirmed with Sanger sequencing. Survival data are from 72 h post-ciprofloxacin treatment ($n = 2$ biological replicates) and a representative experiment is shown for HGA quantification. (**B**) Time-kill assays comparing wild-type and Δ*gndRX* strains treated with ciprofloxacin at different growth phases. Cultures were grown overnight to either early ($OD_{600} = 1$), late ($OD_{600} = 3$), and stationary ($OD_{600} = \sim 4$) growth phases prior to drug treatment. Data are from $n = 2$ biological replicates. (**C**) Principal coordinate plot comparing transcriptomes of wild-type and Δ*gndRX* strains grown to early and late exponential phase prior to RNA extraction and sequencing ($n = 2$ biological replicates for each strain + growth phase combination). Data information: In (**A**, **B**), data are presented as the mean (averaged for clarity) ± SEM. The limit of detection on all applicable plots is indicated with a dashed gray line.

© The Author(s)
